# Interdomain Contacts and the Stability of Serralysin Protease from *Serratia marcescens*

**DOI:** 10.1371/journal.pone.0138419

**Published:** 2015-09-17

**Authors:** Liang Zhang, Anneliese J. Morrison, Patrick H. Thibodeau

**Affiliations:** Department of Microbiology and Molecular Genetics, University of Pittsburgh, School of Medicine, Pittsburgh, PA 15219, United States of America; Tsinghua University, CHINA

## Abstract

The serralysin family of bacterial metalloproteases is associated with virulence in multiple modes of infection. These extracellular proteases are members of the Repeats-in-ToXin (RTX) family of toxins and virulence factors, which mediated virulence in *E*. *coli*, *B*. *pertussis*, and *P*. *aeruginosa*, as well as other animal and plant pathogens. The serralysin proteases are structurally dynamic and their folding is regulated by calcium binding to a C-terminal domain that defines the RTX family of proteins. Previous studies have suggested that interactions between N-terminal sequences and this C-terminal domain are important for the high thermal and chemical stabilities of the RTX proteases. Extending from this, stabilization of these interactions in the native structure may lead to hyperstabilization of the folded protein. To test this hypothesis, cysteine pairs were introduced into the N-terminal helix and the RTX domain and protease folding and activity were assessed. Under stringent pH and temperature conditions, the disulfide-bonded mutant showed increased protease activity and stability. This activity was dependent on the redox environment of the refolding reaction and could be blocked by selective modification of the cysteine residues before protease refolding. These data demonstrate that the thermal and chemical stability of these proteases is, in part, mediated by binding between the RTX domain and the N-terminal helix and demonstrate that stabilization of this interaction can further stabilize the active protease, leading to additional pH and thermal tolerance.

## Introduction

*Serratia marcescens* is an opportunistic human pathogen associated with multiple modes of infection, including pneumonia, empyema, urinary tract infection, corneal keratitis, meningitis, endocarditis, and septic arthritis.[1] As with other pathogens, *S*. *marcescens* secretes multiple virulence factors to promote adherence, alter the host immune and clearance responses, and to facilitate infection. These include extracellular nucleases, chitinases, proteases and lipases and contribute to host immune responses and tissue damage.[1,2] Previous studies have shown that the serralysin metalloprotease, PrtS, is one of the most abundant extracellular virulence factors produced by *S*. *marcescens*.[3,4] Expression of serralysin is associated with corneal keratitis and pneumonia.[5–7]

Serralysin belongs to the zinc-dependent metazincin superfamily and defines the serralysin subfamily, which also includes alkaline protease, AprA, from *Pseudomonas aeruginosa*, and more than 100 other proteases found in gram-negative bacteria.[8–11] Structurally, this family of proteases is highly similar and is comprised of several conserved features. The core architecture of these proteins includes an N-terminal helix or helices, a protease domain, which includes the active-site motif (HEXXHXXGXXH), and an aspartate/glycine-rich Repeats-in-ToXin (RTX) domain at the C-terminus.[10,12,13]

The C-terminal RTX domains define a larger class of enzymatically distinct virulence factors.[14] The RTX domain is comprised of multiple nonapeptide repeats that coordinate Ca^2+^ binding in the native state.[15,16] Structural studies of RTX-containing proteins demonstrate that the conserved aspartate/glycine-rich repeats fold into a β-helix, fully enclosing the Ca^2+^ structural cofactors and coordinating their binding via interactions with the aspartate sidechain carboxylate and glycine backbone carbonyl groups.[12,13,17–19] In addition to the proteases, a variety of important virulence factors, including α-hemolysin (HlyA) from *E*. *coli*, adenylate cyclase toxin (CyaA) from *Bordetella pertussis*, and leukotoxins from *Actinobacillus actinomycetemcomitans* and *Pasteurella haemolytica*, are regulated by Ca^2+^ binding within their RTX domains.[14,20–22]

Previous studies on AprA from *P*. *aeruginosa* and CyaA from *B*. *pertussis* demonstrate Ca^2+^-binding to the nonapeptide repeats regulates the folding of the RTX domains.[13,16,17,23] In the absence of Ca^2+^, the RTX domains remain in an extended conformation. Calcium binding to this domain induces the large disorder-to-order transition and facilitates folding to the native state. In addition, work with AprA demonstrates that folding of the RTX domain nucleates the folding of the protease domain, providing a means to regulate protease structure and function.[13] This work further showed that structural interactions between the N-terminal helix and the folded RTX domain result in stabilization of the native state, though this interaction is not required for efficient folding. The high stability of these proteases is likely beneficial for the pathogen as the enzyme is secreted into an uncontrolled environment. Due to their high stability and activities, these proteases have been used extensively in industrial applications and are functional across a wide range of pHs and show resistance to a variety of solvents. [24]

Given the structural homology between PrtS and AprA, we investigated the role of Ca^2+^ in the regulation of protease folding and native state stability utilizing purified PrtS from *S*. *marcescens*.[10,18,25,26] Calcium binding resulted in the folding and activation of the protease across a variety of conditions. To further elucidate the role of the long-range structural interactions within the serralysin proteases, a series of paired cysteine residues were engineered into the N-terminal helix and the RTX domains at sites predicted to form disulfide bonds in the native state. Formation of the disulfide bonds resulted in further stabilization of the native state and increased protease activity at elevated reaction pH and temperature. These data demonstrate that the interactions between the N-terminal helix and its C-terminal binding site are critical for the high stability of the native state and can be further engineered to provide additional native state protease activity.

## Materials and Methods

### Protein expression and purification

*Serratia marcescens* genomic DNA was used to PCR amplify the open reading frame of PrtS and the PrtS RTX domain (residues 253–471). The PrtS ORF was subcloned into a pBad vector for arabinose induced expression and the RTX domain was subcloned into the pET-DUET vector for T7-regulated expression. Both vectors included 6x-His tags for downstream purification. PCR-based site directed mutagenesis was used to introduce cysteine mutants (A8C-V339C and L12C-R302C). All clones were verified by automated DNA sequencing. The RTX domain boundary was established using the serralysin crystal structure: PDB 1SAT.[10] Residue numbering is consistent with that described in the 1SAT PDB file.

The 6xHis-tagged PrtS full-length proteins and the PrtS-RTX domain expressed at high levels and were purified under denaturing conditions from inclusion bodies using guanidine hydrochloride as previously described.[13,27] Briefly, overnight donor cultures were used to inoculate expression cultures grown in LB media at 37°C. Protein was induced with 1mM IPTG or 0.2% arabinose when the OD_600_ reached ~0.5–0.6. Cells were harvested after 4–5 hours expression at 37°C. Pellets were resuspended into lysis buffer (50 mM Tris, 150 mM NaCl, 5 mM CaCl_2_, pH 6.8), sonicated, and centrifuged at 15,000 RCF for 20 minutes. The insoluble material was resuspended and solubilized in buffers containing 6 M guanidine HCl (GuHCl) (50 mM Tris, 150 mM NaCl, 6 M GuHCl, pH 6.8) and clarified by centrifugation at 40,000 RCF for 30 minutes. The supernatant fraction was then used for purification. For PrtS-RTX, the column was washed in lysis buffer supplemented with 30 mM imidazole and protein was eluted in lysis buffer containing 400 mM imidazole. For full length PrtS protease, the column was similarly washed and eluted with buffers also containing 6M GuHCl. The eluted proteins were concentrated and loaded onto a desalting column (GE Healthcare) in buffers lacking imidazole. Purified RTX protein was flash frozen and stored at -80°C. Full-length protease was stored in GuHCl at 4°C.

### PrtS Refolding

The purified proteins were refolded by rapid dilution into cold buffer (50 mM Tris, 150 mM NaCl, 0–20 mM CaCl_2_, pH 6.8) on ice for 15 minutes prior to activity and/or spectroscopic measurements.[13] Misfolded or unfolded proteins were removed from the refolding reactions by centrifugation and/or filtration through a Microcon YM-100 filter (Millipore). For stability experiments, the wild type PrtS and mutants (A8C-V339C or L12C-R302C) were refolded in the presence and absence of disulfide enhancer (NEB) or in the presence of 1 mM dithiothreitol (DTT). For maleimide labeling, protein in GuHCl was incubated in the presence of 10-20x excess molar concentration of maleimide (Sigma).

### Analytical Gel Filtration Chromatography

Gel filtration chromatography was accomplished as previously described using a Superdex 75 300GL size exclusion column (GE Healthcare) at 4°C.[13] Protein samples were assessed in the presence and absence of Ca^2+^ after column equilibration with refolding buffer.

### Intrinsic Protein Fluorescence

Fluorescence emission spectra of the PrtS-RTX domain were collected on a BioTek Synergy 4 multimode plate reader as previously described.[13] Protein was titrated with 0–20 mM Ca^2+^. Emission spectra from 300–400 nm were collected with an excitation wavelength of 280 nm.

### Protease Assay

Protease activity was evaluated using a BODIPY-conjugated casein substrate (EnzChek, Invitrogen) and MMP fluorescent peptide substrates (Mca-Lys-Pro-Leu-Glg-Leu-Dpa-Ala-Arg-NH2. Mca: (7-Methoxycoumarin-4-yl)acetyl; Dpa: N-3-(2, 4-Dinitrophenyl)-L-2,3-diaminopropionyl) (R&D systems) or DNP-Pro-Leu-Gly-Met-Trp-Ser-Arg-OH (Calbiochem).[13] Cleavage of the peptide results in dequenching of the fluorophore and an increase in fluorescence. Fluorescence emission was measured on a BioTek Synergy 4 multi-mode plate reader in kinetic or endpoint modes. For pH stability experiments, refolded protein was diluted in Bis-Tris propane buffer at multiple pH values (50 mM Bis-Tris propane, 150 mM NaCl, 2 mM CaCl_2_). For temperature stability experiments, refolded proteins were incubated at the specified temperature for 10 minutes prior to addition of the reaction buffers and substrate. The assay was then incubated for 10 minutes at temperature before 1 mM EDTA was added to quench the proteolytic reactions.

### Western blotting

Polyclonal α-RTX antibody, raised against the *Pseudomonas aeruginosa* alkaline protease RTX domain showed cross-reactivity to the PrtS-RTX sequences and was used to detect PrtS or PrtS-RTX proteins.[13]

### Data Analysis

Data were fit using a four-parameter Hill equation. Non-linear regression was performed using SigmaPlot. Data presented were collected from at least two independent protein purifications with *n> = 3* for all experiments.

## Results

### Calcium-induced folding of PrtS-RTX

**Fig 1:**
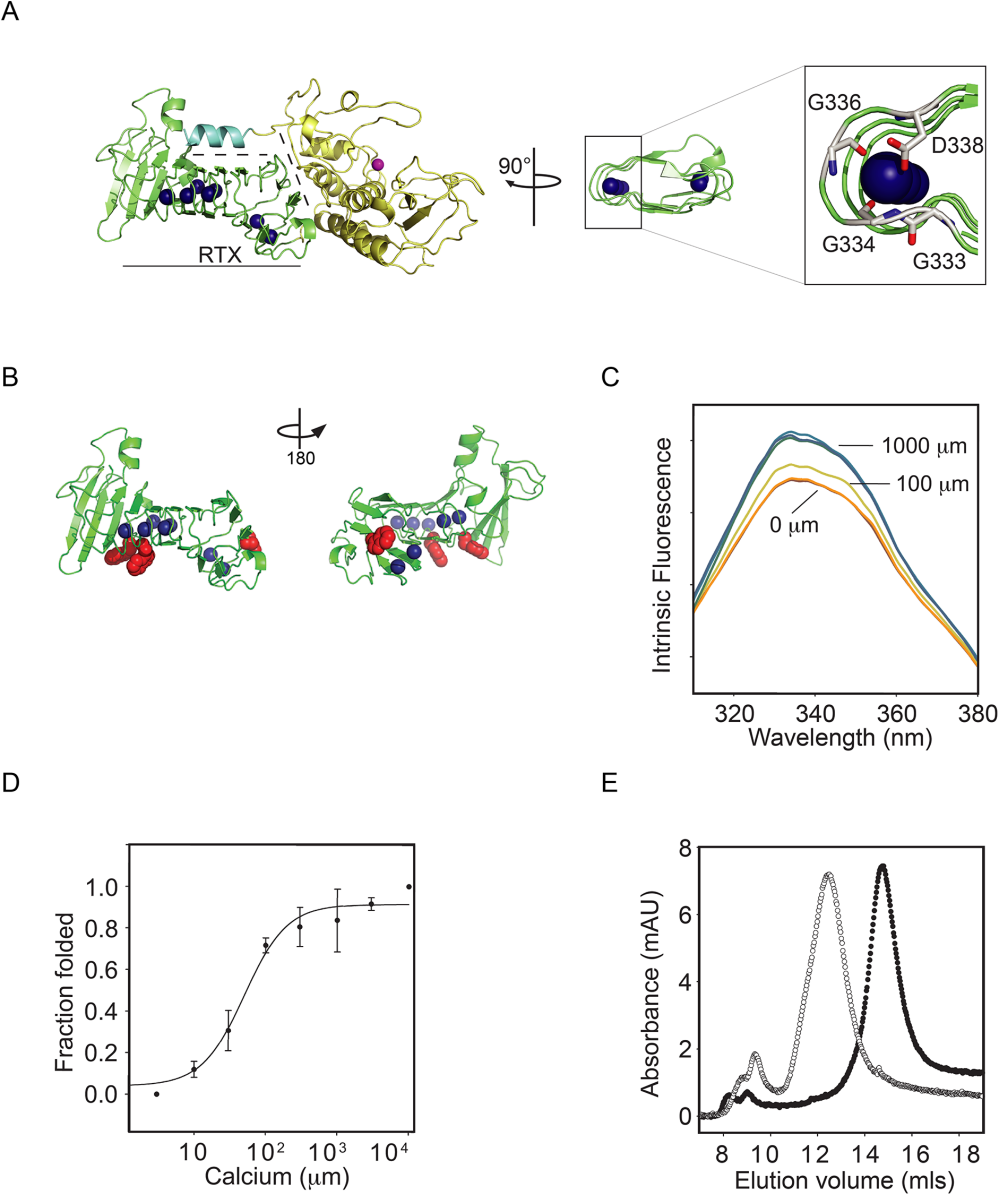
Calcium-induced PrtS-RTX folding. The folding of the purified PrtS RTX domain was assessed biophysically as a function of calcium binding. *A*, a cartoon showing crystal structure of the PrtS protein is shown (based on the 1SAT PDB file).[10] The RTX domain is shown (green) separated by dashed lines from the protease domain (yellow) and the N-terminal helix (cyan). The blue spheres represent calcium ions that are bound within the structure and the active site zinc is shown in magenta. A close-in view of the bound calcium is shown on the right, coordinated within the RTX repeat sequence. *B*, a cartoon representation of the RTX domain is shown rotated about the vertical axis by 180°. The sidechains of the three native tryptophan residues are shown in red. *C*, representative tryptophan fluorescence emission spectra of PrtS-RTX in various calcium concentrations are shown. The normalized fluorescence intensity increases with increasing Ca^2+^ concentrations. Select calcium concentrations are labeled for clarity. *D*, the calcium-binding isotherm for PrtS-RTX is shown as a function of fluorescence intensity change and Ca^2+^ concentration. Data shown are mean ± standard deviation. *E*, representative analytical gel filtration chromatographs show the hydrodynamic states of apo (*open circle*) and Ca^2+^-bound (*closed circle*) PrtS-RTX protein.

Previous work on AprA from *Pseudomonas aeruginosa* and CyaA from *Bordetella pertussis* demonstrated that Ca^2+^ induces the folding of RTX domains, which can then nucleate the folding of other protein domains.[13,17,28] Based on previous folding studies, the RTX domain was purified and evaluated in isolation from the N-terminal domains (N-terminal helix and protease domain).[13] From these studies, three key features of the protease RTX domain emerge (Fig 1A). First, Ca^2+^-binding regulates the folding of the RTX domain. Second, interactions across the RTX-protease domain interface nucleate protease domain folding and protease activation. Finally, interactions with the N-terminal helix and the folded RTX domain stabilize the native state of the protease.

Intrinsic protein fluorescence was used to monitor RTX structure as a function of Ca^2+^ binding. The PrtS-RTX contains three tryptophan resides that were used as structural probes (Fig 1B). Emission spectra were collected for each concentration in the Ca^2+^ titration and emission intensity was utilized as a probe for RTX domain folding. In both the presence and absence of Ca^2+^, the spectra showed peak intensities at 332 nm. The addition of Ca^2+^ resulted in increased fluorescence intensities with only subtle changes in peak wavelength, consistDent with the partial surface exposure of the tryptophan probes in the native state. (Fig 1C). The subtle wavelength changes could be seen as a loss of a spectral shoulder at roughly 350 nm. This increase in fluorescence intensity was non-linear with Ca^2+^ addition and appeared to saturate at concentrations greater than 100–200 μM. The increase in intensity at 332 nm correlated with the loss of the minor shoulder at 350 nm. The increase of fluorescent intensity at 332 nm and loss of the 350 nm shoulder indicates that the tryptophan residues were responding to Ca^2+^ binding. These changes were not apparent when the protein was incubated in the presence of other divalent cations, including both Mg^2+^ and Mn^2+^ (data not shown).

Calcium binding was assessed as a function of the fluorescence intensity at 332 nm. When the fluorescence intensity was plotted against [Ca^2+^], the binding isotherms appeared sigmoidal. Below 10 μM Ca^2+^, intrinsic fluorescence was low and did not vary with changes in Ca^2+^ concentration. Between 10 and 200 μM [Ca^2+^], fluorescence increased and appeared to saturate between 100–200 μM [Ca^2+^] (Fig 1D). Nonlinear regression was utilized to fit the fluorescence data to the Hill equation with the apparent K_d_ = 51 ± 7 μM and a Hill coefficient of 1.4 ± 0.2.

The structural changes associated with Ca^2+^ binding were then assessed using analytical gel filtration. In the absence of Ca^2+^, the RTX domain eluted as a single predominant peak at 12.5 mls (Fig 1E, *open circles*). A small population of the protein also eluted in the void and likely contained aggregates of the RTX protein. The Ca^2+^-bound protein was similarly injected onto a column that was pre-equilibrated with 2 mM Ca^2+^. In the presence of Ca^2+^, the RTX domain eluted at approximately 14.7 mls (Fig 1E, *closed circles*). Neither peak showed significant signs of tailing or asymmetry. The shift in elution volume was consistent with a disordered and extended RTX polypeptide in the absence of Ca^2+^ and a compact, folded RTX domain in the presence of 2 mM Ca^2+^. Based on known gel filtration standards, the hydrodynamic radius of the Ca^2+^ bound protein was in close agreement with the predicted molecular mass of roughly 25 kDa.

### Calcium-induced folding of PrtS

**Fig 2:**
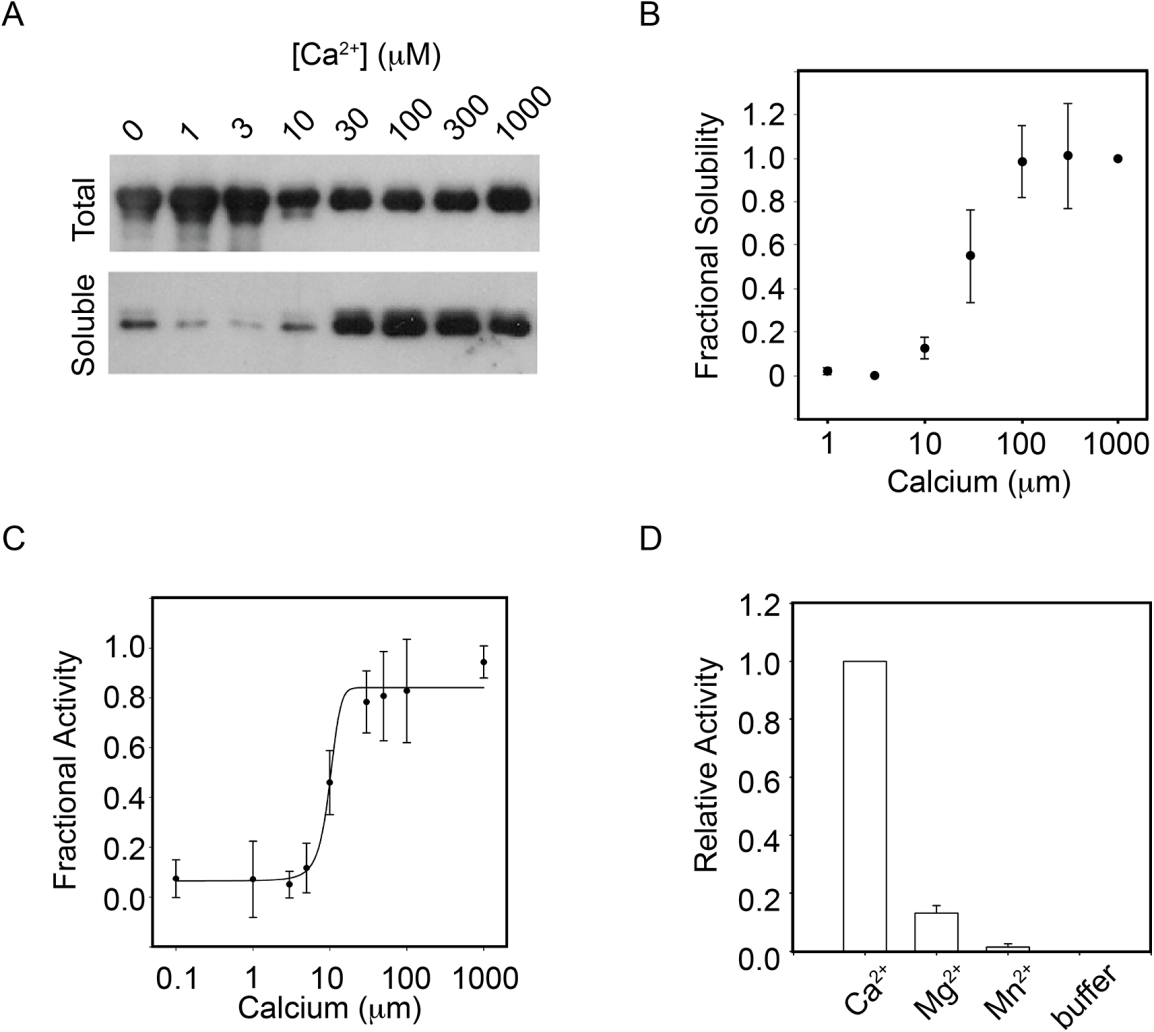
Ca^2+^-induced PrtS folding. The folding and activation of purified full-length PrtS was evaluated as a function of calcium binding. *A*, a representative western blot showing the calcium mediated refolding is shown. Protein was refolded in increasing calcium and filtered using a 100 kDa MWCO filter. The prefiltered samples are shown (*Total*) as a loading control with the eluate (*Soluble*) representing the compact, folded protein. *B*, densitometric analyses of the soluble fraction of PrtS are shown after refolding. The integrated band intensities are plotted against [Ca^2+^]. *C*, protease activity was assessed as a function of calcium in the refolding buffer using an enzymatic assay. Fractional activity is plotted against calcium concentration and fit using the Hill equation. *D*, the specificity of the PrtS refolding reaction was assessed using Mg^2+^ and Mn^2+^ cations. Endpoint fluorescence activity assays are shown for Ca^2+^, Mg^2+^ and Mn^+^ in conditions where Ca^2+^ is saturating. Data shown are mean ± standard deviation from at least three independent experiments.

To evaluate Ca^2+^-induced folding of the full-length PrtS protein, recombinant PrtS was purified under denaturing conditions and refolded *in vitro*. Protein solubility and monodispersity were utilized to assess the structural changes associated with Ca^2+^ binding, as previously described.[13] PrtS refolding reactions were performed with Ca^2+^ concentrations to 2 mM. Following incubation, an aliquot of the protein was removed from the reaction and used as an input control for each refolding condition (Fig 2A, *Total*). The remaining protein was then clarified using a Microcon YM-100 filter to remove aggregates and other unfolded protein species. The eluate was then assessed by western blotting and activity assays. In < 10 μM Ca^2+^, the PrtS protein failed to elute from the YM-100 column, consistent with protein aggregation or misfolding. As Ca^2+^ concentrations increased between 10–100 μM, the soluble fraction of PrtS increased (Fig 2A, *Soluble*). Above ~100 μM Ca^2+^, the soluble fraction appeared to saturate and no further increase in soluble protein could be detected with increasing [Ca^2+^]. Densitometric analyses of the western blots confirmed a cooperative Ca^2+^ dependence of PrtS refolding with an apparent affinity of 27 ± 11 μM with a Hill coefficient of 1.7 ± 0.4 (Fig 2B). Under saturating conditions, greater than 80% of the input protein was folded *in vitro*, as assessed by quantification of the total and soluble fractions by western blotting using the α-RTX antibody.

Similar Ca^2+^ titrations were performed and assessed using a fluorescent protease activity assay. Here, cleavage of the fluorescent peptide by an active protease results in increased fluorescence and can be followed kinetically or as a reaction endpoint. The PrtS protease was refolded in increasing Ca^2+^ concentrations and the mean steady state reaction velocity was measured kinetically (Fig 2C). In < 10 μM Ca^2+^, the refolded protein showed little protease activity against the fluorescent peptide substrates. As Ca^2+^ concentrations increased between 10–100 μM, protease activity increased with Ca^2+^. Above 100 μM Ca^2+^, the steady state activity saturated. These observations were roughly consistent with those seen in the solubility experiments using the full-length protein and the tryptophan fluorescence experiments using the RTX domain. Nonlinear regression was used to fit the plot of Ca^2+^ versus protease activity using the Hill equation. The apparent K_d_ for Ca^2+^-induced activation of PrtS was 14 ± 6 μM with a Hill coefficient of 1.6 ± 0.5. To investigate whether the folding of the protein is specific for Ca^2+^, two other divalent ions (Mg^2+^ and Mn^2+^) were tested using the activity assay. Endpoint fluorescence after incubation in the presence of 2 mM divalent cations was used to assess their ability to fold and activate the protease (Fig 2D). Under these conditions, robust protease activity was detected in the presence of Ca^2+^, as seen in the dose response experiments. In the presence of 2 mM Mg^2+^, roughly 13% activity was seen, as compared to protease in the presence of 2 mM Ca^2+^. With refolded in 2 mM Mn^2+^, no significant protease activity was detected. This demonstrates a preferential binding for Ca^2+^ over other divalent cations in the full-length protein.

### Stability of PrtS

**Fig 3:**
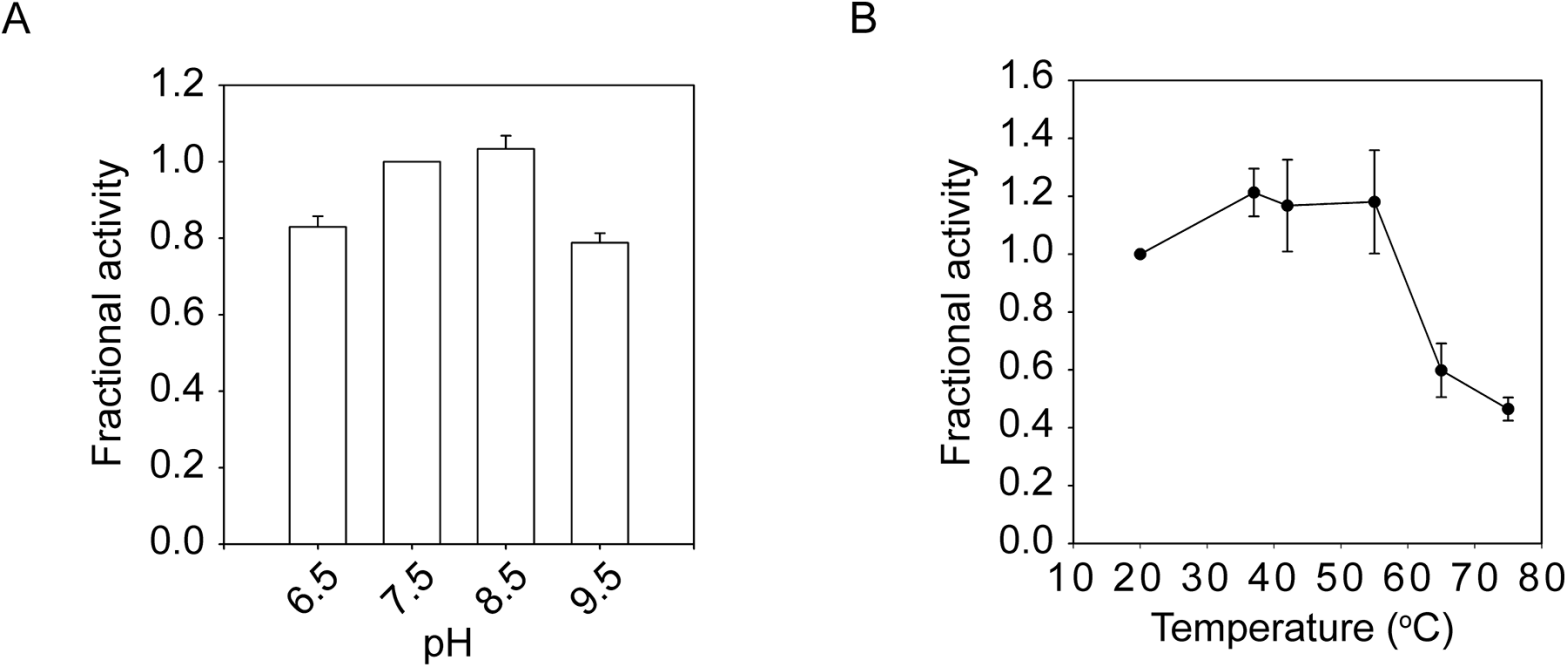
pH and temperature stability of PrtS. The relative pH and thermal sensitivities of the refolded PrtS were assessed using functional assays. *A*, a bar graph showing the relative activity of PrtS between pH 6.5 and 9.5 is shown. Activity was normalized to that seen at pH 7.5. *B*, the relative activity of PrtS between 22–75°C is shown. Protease activity was normalized to that seen at 22°C. Data shown are mean ± standard deviation from at least three independent experiments.

Many bacterial proteases are known to maintain high activity under a wide range of physical conditions (pH, temperature, solvent).[13,14,29] To evaluate the pH stability of the refolded PrtS, protease activity was assessed at pHs between 6.5 to 9.5 using the fluorescent peptide protease assay. After refolding in the presence of 2 mM Ca^2+^, the PrtS protease was active under a wide range of pH conditions (Fig 3A). When normalized to PrtS activity at pH 7.5, protease activity peaked at pH 8.5 with roughly 120% of the activity seen at pH 7.5. The protein showed roughly 80% activity at pH 6.5 and 75% activity at pH 9.5, as compared to that seen at pH 7.5. These data are consistent with previous reports of maximal protease activity under mildly alkaline conditions for this family of proteases.[13,30]

The activity of PrtS was also assessed over a range of reaction temperatures using the fluorescence activity assay. Protease refolding was accomplished under saturating Ca^2+^ and the refolded protease was subsequently incubated at the indicated temperatures in the presence of the fluorescent substrate. After equilibration for 10 minutes at reaction temperatures, the peptide was added and the digests proceeded for 10 minutes before being quenched with EDTA. Endpoint fluorescence of the cleaved peptide was then measured (Fig 3B). The fluorescence signals were normalized to control reactions at 22°C. Protease activity plateaued between 37°C and 55°C and was 120–125% that of samples incubated at 22°C. Above 55°C, protease activity fell with increasing reaction temperature. At 65°C, the observed protease activity was 60% that seen at 22°C. At 75°C, protease activity fell to roughly 50% of the room temperature control. These data were similar to those determined for protein purified directly from culture supernatants and suggest that the refolded protease has biochemical properties similar to that purified under native conditions.

### Stabilization of PrtS by domain-domain interactions

Our previous work on AprA demonstrated that protease stability arises, in part, from the interaction between the N-terminal helix and a saddle formed on the side of the folded RTX domain.[13] When disrupted by either single site substitutions or by deletion of the N-terminal helix, the stability of AprA was reduced in thermal and chemical denaturation experiments. Mutations within the N-terminal helix disrupted protease activity at elevated temperature and decreased the conformational stability of the protease in response to chemical denaturation experiments.

**Fig 4:**
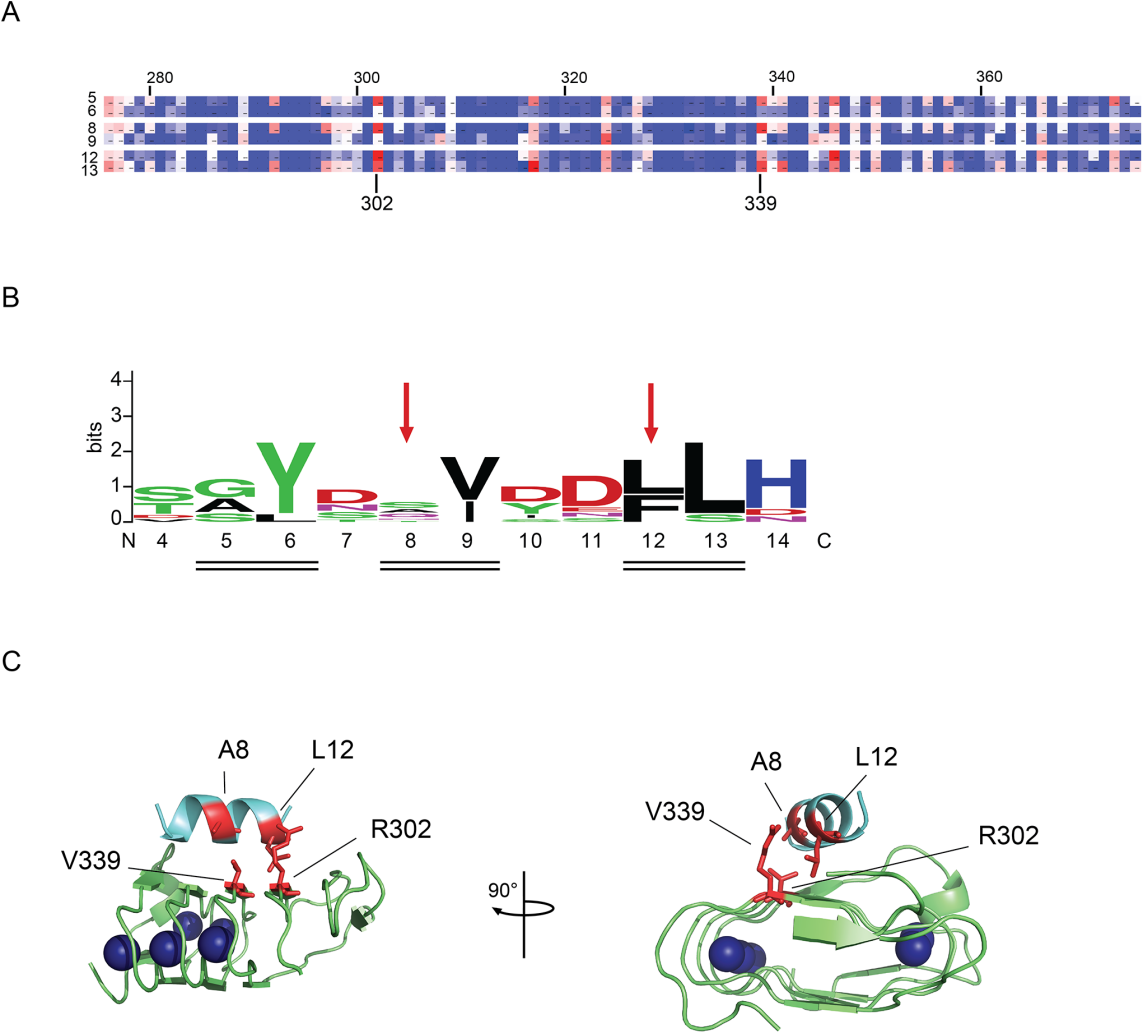
Domain-domain interactions in PrtS. Paired cysteine residues were introduced into the RTX domain and the N-terminal helix based on covariance analysis of the serralysin family and the strucures of PrtS and *Pseudmonas aeruginosa* alkaline protease (PDB code 1SAT and 1KAP).[10,18] *A*, a heatmap of the covariance between the N-terminal helical residues and the folded RTX domain is shown. High covariance is colored red and low covariance is colored in blue. Residues in the N-terminal helix facing away from the RTX domain are omitted for clarity and show little covariance with the RTX domain. Residues are numbered according to the 1SAT PDB file on the left and top of the heatmap. The specific positions of R302 and V339 are indicated on the bottom of the heat map. *B*, a sequence logo of the N-terminal helix sequence from the serralysin family of protease is shown and numbered according to the PrtS structure. Red arrows show the positions of the cysteine substitutions in the N-terminal helix. The double lines indicated residues that are in direct contact with the folded RTX domain. *C*, a cartoon shows the N-terminal helix (*cyan*) packed against the RTX domain (*green*) in two views rotated about the vertical axis by 90°. Sites chosen for cysteine substitution are shown (*red*). The cysteine pairs are: A8-V339 and L12-R302. The bound calcium ions are shown as blue spheres.

Statistical co-variance analyses of known RTX proteases were utilized to ascertain how conserved these paired interactions are across the family of proteases and to identify residues that are evolutionarily coupled to the sequences of the N-terminal helix. Sequence covariance between the N-terminal helix sequences and the core residues of the RTX domain were performed using the Direct Coupling Analysis Workbench.[31] These analyses showed clustered covariance between the N-terminal helix and residues in the core of the RTX domain (Fig 4A). The covariance analyses demonstrated that several specific interactions were evolutionarily preserved and likely contributed to the interactions between the N-terminal helix and RTX domain. These interactions showed a periodicity that indicated covariance was increased on the helical face that docked against the RTX domain. Covariance was highest between the N-terminal helix and residues Arg302 and Val339 along this domain-domain interface, though covariance was also seen with regions of the RTX not in direct contact with the N-terminal helix in the native structure (Lys317, Ala324, Asn346, Val348, K350).

Residues on the helical face docked against the RTX domain (positions 5, 6, 8, 9, 12, 13) were assessed for their conservation and their potential interactions with Arg302 and Val339. Multiple sequence analyses indicated that many sites showed sequence variation within this amphipathic helix and suggested that substitutions might be tolerated at positions Ala8 and Leu12 (Fig 4B).[32] Analysis of available RTX protease structures were consistent with the covariance analysis and suggested that residues Arg302 and Val339 in the RTX domain made contacts with residues Ala8 and Leu12 in the N-terminal helix (Fig 4C).

Based on the sequence and structural analyses, a series of cysteine substitutions were selected to assess whether stabilization of this domain-domain interaction could be used to further stabilize the protease. Paired cysteine substitutions at positions A8C-V339C and L12C-R302C were introduced by site directed mutagenesis (Fig 4C). The effects of these substitutions and the predicted disulfides formed by the paired cysteine residues were assessed using the fluorescence-based peptide digestion assay in the presence of buffer additives that promote or inhibit disulfide formation.

**Fig 5:**
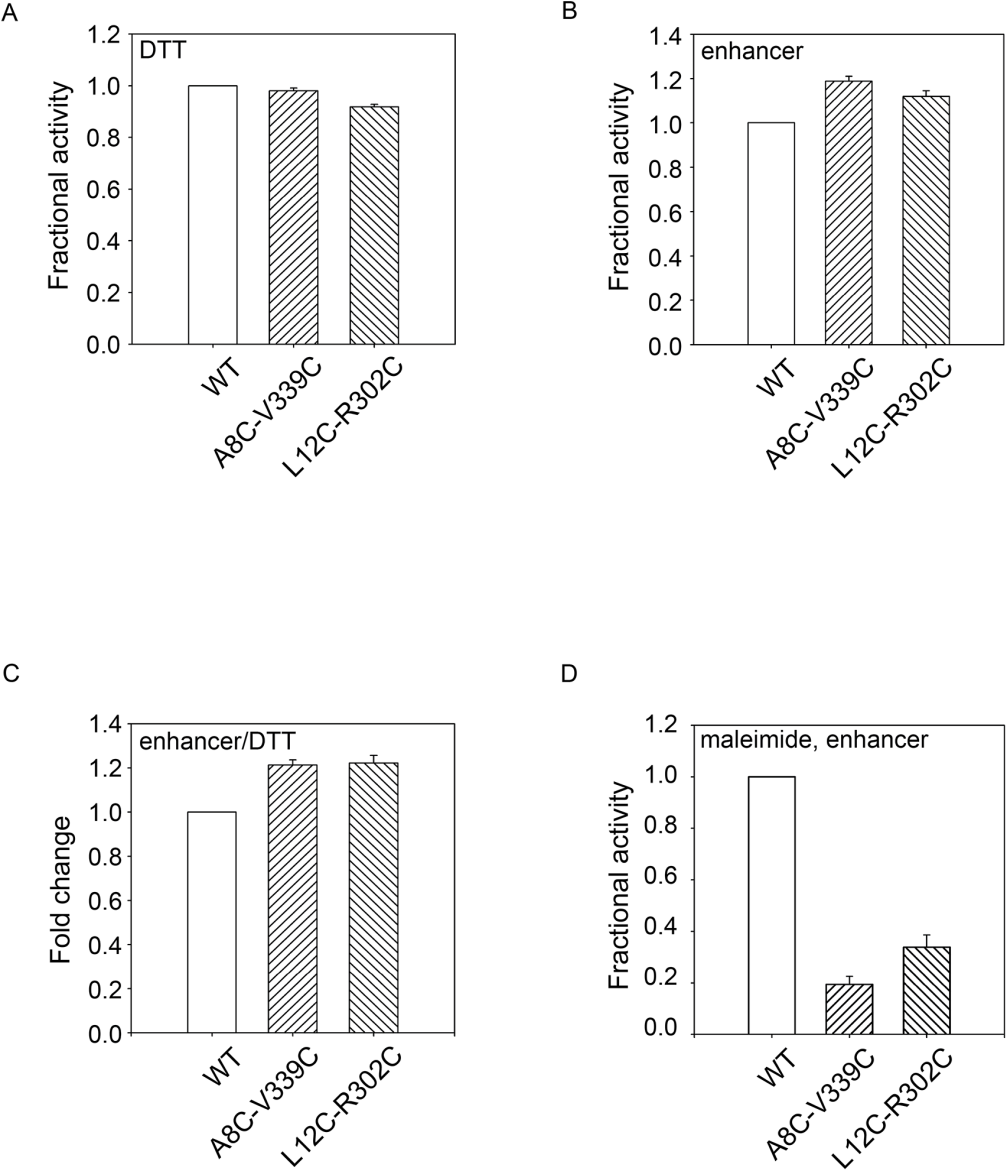
Disulfide stabilization under relaxed biochemical conditions. The PrtS cysteine mutants were refolded and assessed functionally in reducing and oxidizing conditions and activity was assessed at pH 7.5 at 55°C. *A*, activity assays of the PrtS proteins refolded in the presence of DTT are shown and normalized to the activity observed with the wildtype protein. *B*, activity assays of the PrtS proteins refolded in the presence of a disulfide-enhancing additive are shown and normalized to the activity observed with the wildtype protein. *C*, the fold change between reducing and oxidizing conditions, *A and B*, is shown for the wildtype and mutant proteins. *D*, the PrtS protein were pre-incubated with maleimide and refolded as in *C*. Activity is shown normalized to the wildtype. Data shown are mean ± standard deviation from at least three independent experiments. Relative activity values were derived from the mean reaction velocities under steady state conditions with wildtype PrtS activity normalized to 1.0.

The proteases were first refolded then diluted into reaction buffers at the indicated temperatures. Activity was first assessed under relaxed conditions where the wildtype protein showed high activity: pH 7.5 and 55°C. In the presence of DTT, small changes in PrtS activity could be seen using the fluorescent peptide substrates (Fig 5A). The mutants showed greater than 90% activity when compared to the wildtype, consistent with proper folding and activation from the DTT-reduced state. When refolded in the presence of a disulfide-promoting additive, the mutant protein showed a modest increase in protease activity when compared to the wildtype under identical conditions (Fig 5B). The A8C-V339C mutant showed a ~20% increase in protease activity while the L12C-R302C mutant showed a ~15% increase in protease activity, as compared to wildtype. The redox dependent changes in mutant protease activity were consistent with structural changes associated with putative disulfide formation by the paired cysteines and resulted in an increase of protease activity of approximately 20–25% (Fig 5C).

To further evaluate how changes in protease activity relate to putative disulfide formation, the denatured proteases were incubated in maleimide to specifically label the engineered cysteine residues. Based on previous work, the disruption of the helix-RTX domain packing by the maleimide-labeled cysteines would be predicted to decrease protease stability and activity at elevated temperatures and would mimic substitutions in the RTX-helical interface seen in AprA. [13] The maleimide adduct introduces a change in the cysteine sidechain volume and would mimic the introduction of a large amino acid substitution at these sites. Pre-incubation of the protease with maleimide resulted in a dramatic loss of protease activity as measured using the fluorescent peptide activity assays and was consistent with a disruption in protease folding and activation by the maleimide modification (Fig 5D). The activity of the A8C-V339C mutant decreased by roughly 80%, while the L12C-R302C mutant decreased by approximately 70%. This loss of protease activity at 55°C is consistent with the disruption of proper domain folding and association and mimicked the single site substitution experiments with AprA. These data are consistent with the observations that these helix-RTX interactions are important for protein stability and are disrupted by the added bulk of the maleimide reagent in this interface.

**Fig 6:**
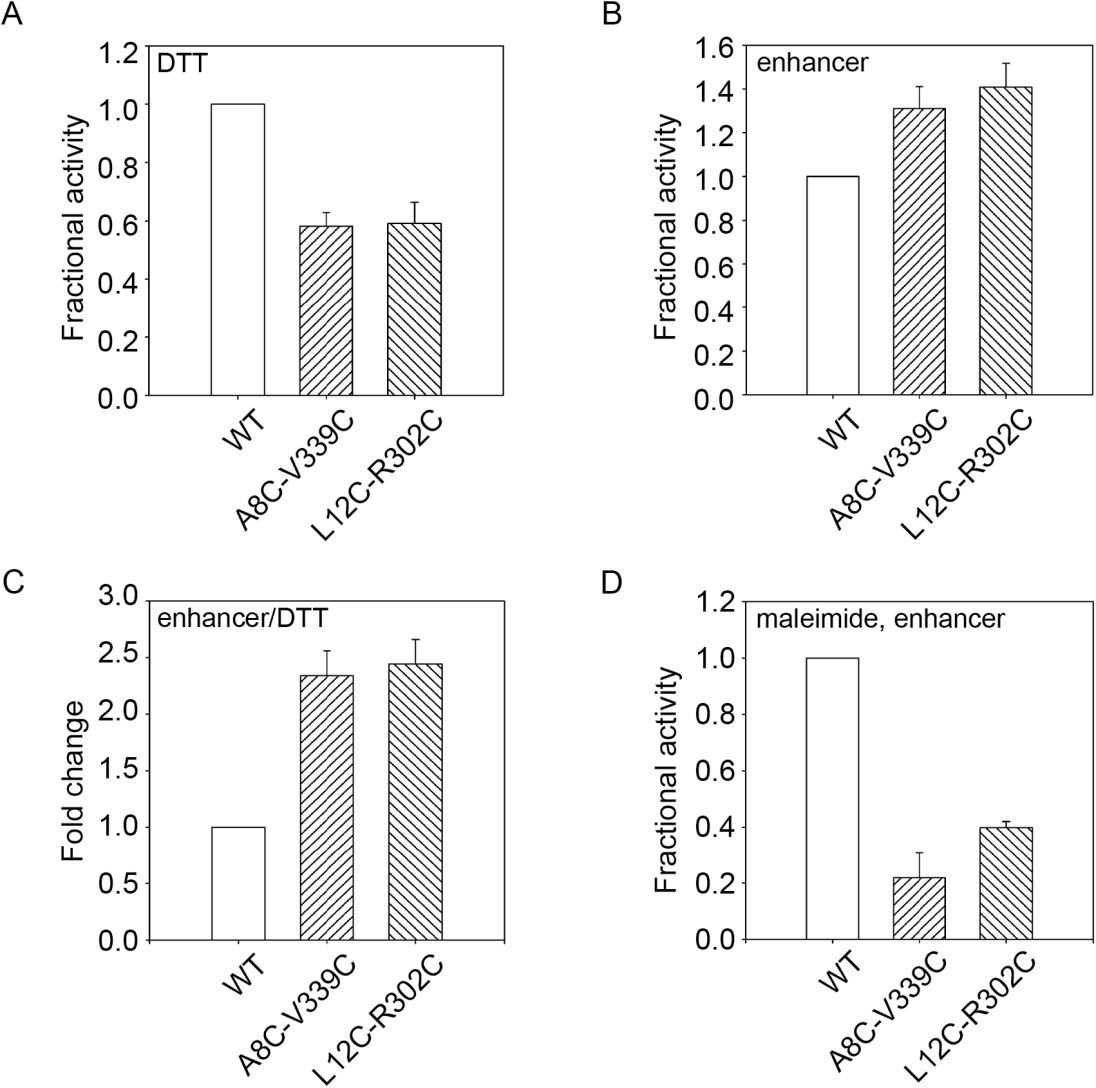
Disulfide stabilization under stringent biochemical conditions. The PrtS cysteine mutants were refolded and assessed functionally in reducing and oxidizing conditions and activity was assessed at pH 9.5 at 55°C. *A*, activity assays of the PrtS proteins refolded in the presence of DTT are shown and normalized to the activity observed with the wildtype protein. *B*, activity assays of the PrtS proteins refolded in the presence of a disulfide-enhancing additive are shown and normalized to the activity observed with the wildtype protein. *C*, the fold change between reducing and oxidizing conditions, *A and B*, is shown for the wildtype and mutant proteins. *D*, the PrtS protein were pre-incubated with maleimide and refolded as in *C*. Activity is shown normalized to the wildtype. Data shown are mean ± standard deviation from at least three independent experiments. Relative activity values were derived from the mean reaction velocities under steady state conditions with wildtype PrtS activity normalized to 1.0.

To further evaluate the effects of putative disulfides, more restrictive reaction conditions, pH 9.5 and 55°C, were selected based on the pH and temperature activity profiles (Fig 3A and 3B). Again, the proteases were refolded in the presence and absence of additives that inhibited or promoted disulfide formation. When refolded in the presence of DTT and assessed at these restrictive conditions, the mutant protease showed a dramatic decrease in total protease activity, as compared to wildtype (Fig 6A). The difference between wildtype and mutant protease activity was greater at pH 9.5 than at pH 7.5, consistent with a more restrictive condition at the elevated pH.

In contrast, refolding in the presence of disulfide-promoting additives increased protease activity in the restrictive conditions when compared to wildtype (Fig 6B). The A8C-V339C mutant showed a 35% increase in total activity while the L12C-R302C mutant showed a 40–45% increase in activity, as compared to wildtype. The redox dependent change in protease activity was 2.3–2.5 fold for both of the cysteine mutant pairs at pH 9.5 (Fig 6C). As seen in more relaxed buffer conditions, pre-labeling with maleimide reduced the mutant proteins’ activity by 70–80%, similar to the reduction seen at pH 7.5 (Fig 6D). These data were consistent with the native state stabilization of PrtS by the engineered disulfides. In addition, the reduced-state instability of the mutants and the maleimide-conjugated proteins provided further evidence that the intrinsic stability of these proteases is, in part, due to the domain-domain interactions between the N-terminal helix and the folded RTX domain.

## Discussion

The structures of the Repeats-in-ToXin virulence factors are dynamic and, at least for the RTX proteases, tightly regulated by Ca^2+^ binding.[13–15,17,28,33] Calcium binding provides for the regulation of the protease during secretion, providing a means to control enzymatic function before secretion and stability after secretion. Based on previous studies evaluating the Ca^2+^-associated regulation of RTX protease structure and function, we sought to further define the physical basis of the RTX protease’s high stability and engineer increased stability.

As with multiple other RTX proteins, the RTX domain of PrtS is dynamic and folds in response to Ca^2+^ binding (Fig 1). The apparent Ca^2+^ affinity, as measured by tryptophan fluorescence, was 51 ± 7 μM and showed positive cooperativity. The affinity measurement was similar to the highly similar AprA RTX domain from *P*. *aeruginosa* which was determined by multiple methods to be 44 ± 13 μM. Differences in measured cooperativity were more apparent between the two proteins with the PrtS RTX protein showing lower apparent cooperativity (Hill coefficient = 1.4 ± 0.2) as compared to AprA (3.1 ± 0.1).[13] Given the high sequence similarity between the RTX sequences of PrtS and AprA (63% identity, 77% similarity), additional comparison of the Ca^2+^-induced folding of these two homologous domains may reveal the basis for the observed cooperative folding and warrants additional investigation.

Consistent with previous studies of AprA from *P*. *aeruginosa*, Ca^2+^ binding to the RTX domain appears to be coupled to the folding and activation of the full-length protease (Fig 2).[13] In the absence of Ca^2+^, the PrtS protease aggregated when diluted from denaturant. Calcium in the refolding buffers blocked this aggregation and simultaneously lead to the production of soluble and active protein. The Ca^2+^-dependence of the full-length protease solubility (27 ± 11 μM) and activity (14 ± 6 μM) showed an apparent affinity for Ca^2+^ that was slightly higher than that measured for the RTX domain alone (51 ± 7 μM). The measured cooperativity values were similar for both the RTX and full-length protease (Hill coefficients = 1.4 ± 0.2 vs 1.6 ± 0.5). The subtle differences in Ca^2+^ affinity in the full-length protein as compared to the purified RTX domain may reflect stabilizing domain-domain interactions or reflect differences in the experimental methodologies. As with the RTX domain, the Ca^2+^-dependence of full-length PrtS folding and activation was similar to that reported for AprA (K_d_ = 60 μM; Hill coefficient = 2.7).[13] Further comparison of these highly similar proteases may reveal the physical basis underlying the differences in apparent Ca^2+^-binding affinity and structural cooperativity.

Similar to many other bacterial exoproteases, PrtS exhibits high stability with robust protease activity across a wide range of temperatures and pHs (Fig 3). The evolution of these properties is likely beneficial for the bacterium as the extracellular environment is unregulated and, once secreted, the bacterium has no means to regulate the activity of the protein. As a result, increased stability likely yields an increased efficiency in nutrient acquisition and pathogen defense.[23,34–37]

Previous studies with AprA demonstrate that, once folded, the saddle of the folded RTX β-helix bound the N-terminal helix.[10,13,18] These contacts are extensive (>600 Å^2^) and, when disrupted, cause a loss in protease stability. As a result, the Ca^2+^ regulated AprA RTX folding is critical for two events: the nucleation of protease domain folding and its activation and the formation of the helical saddle that provides for protease stability. In the absence of the N-terminal helix or disruption of its packing to the RTX domain, protease stability and activity are severely compromised. Coupling these two functions to RTX folding is likely beneficial for protease secretion, which is thought to require an unfolded and extended polypeptide.[14,38] In the low Ca^2+^ of the intracellular environment, the protease remains unfolded. Only after being secreted in a Ca^2+^-rich environment does the protease adopt native structure. Once in the native state, the N- and C-terminal structures are competent to bind and stabilize the folded form.

Building on this model, the introduction of cysteine pairs resulted in the hyper-stabilization of the PrtS protease in a disulfide-dependent fashion (Figs 4–6). The introduction of the paired cysteine residues had minimal effects on protease activity in reducing conditions in relaxed conditions, suggesting that the cysteine mutants could fold to a functional state. Conditions that promoted disulfide formation increased protease activity under relaxed and restrictive conditions, consistent with the formation of a disulfide that tethered the N-terminal helix to the RTX domain and stabilization of the native state. Disrupting these native interactions decreased protease thermostability. Under restrictive conditions, the paired disulfides reduced total protease activity in the reduced state, consistent with changes in domain-domain packing associated with these substitutions. Similarly, maleimide labeling decreased protease activity under both relaxed and restrictive conditions. These results are consistent with mutagenic studies of AprA wherein disruption of the RTX-helical interface decreased protease stability and thermotolerance and suggest that PrtS is similarly stabilized by these domain-domain contacts.[13]

These data provide additional evidence for the physical basis of RTX protease stability and suggest means by which these activities could be modulated for a variety of purposes. Disruption of the RTX-N-terminal helix interaction may provide a mechanism to down-regulate protease stability and, in turn, activity. Such an approach might be useful in designing modulators to decrease the activity of these virulence factors therapeutically. In contrast, stabilization of this interaction provides a means to up-regulate protease stability and activity and may be useful for industrial applications wherein enzymatic function is required at elevated temperatures, non-neutral pHs or under harsh conditions.
